# Risk of discontinuation of Advanced Therapy Medicinal Products clinical trials

**DOI:** 10.3402/jmahp.v4.32232

**Published:** 2016-08-02

**Authors:** Eve Hanna, Cecile Rémuzat, Pascal Auquier, Mondher Toumi

**Affiliations:** 1 Faculty of Medicine – Public Health department, University Aix-Marseille, Marseille, France; 2 Creativ-Ceutical, Paris, France

**Keywords:** advanced therapy medicinal products, ATMP, clinical trials, risk of discontinuation, RCT

## Abstract

**Objective:**

Advanced therapy medicinal products (ATMPs) constitute a class of innovative products that encompasses gene therapy, somatic cell therapy, and tissue-engineered products (TEP). There is an increased investment of commercial and non-commercial sponsors in this field and a growing number of ATMPs randomized clinical trials (RCT) and patients enrolled in such trials. RCT generate data to prove the efficacy of a new therapy, but the discontinuation of RCTs wastes scarce resources. Our objective is to identify the number and characteristics of discontinued ATMPs trials in order to evaluate the rate of discontinuation.

**Methods:**

We searched for ATMPs trials conducted between 1999 to June 2015 using three databases, which are Clinicaltrials.gov, the International Clinical Trials Registry Platform (ICTRP), and the EU Drug Regulating Authorities Clinical Trials (EudraCT). We selected the ATMPs trials after elimination of the duplicates. We identified the disease areas and the sponsors as commercial or non-commercial organizations. We classified ATMPs by type and trial status, that is, ongoing, completed, terminated, discontinued, and prematurely ended. Then, we calculated the rate of discontinuation.

**Results:**

Between 1999 and June 2015, 143 withdrawn, terminated, or prematurely ended ATMPs clinical trials were identified. Between 1999 and June 2013, 474 ongoing and completed clinical trials were identified. Therefore, the rate of discontinuation of ATMPs trials is 23.18%, similar to that for non-ATMPs drugs in development. The probability of discontinuation is, respectively, 27.35, 16.28, and 16.34% for cell therapies, gene therapies, and TEP. The highest discontinuation rate is for oncology (43%), followed by cardiology (19.2%). It is almost the same for commercial and non-commercial sponsors; therefore, the discontinuation reason may not be financially driven.

**Conclusion:**

No failure risk rate per development phase is available for ATMPs. The discontinuation rate may prove helpful when assessing the expected net present value to support portfolio arbitration and may be considered by patients and potential investigators in their decisions to participate in ATMP trials. These results carry limitation because the rationale for discontinuation is unknown. Further research about the reasons of discontinuation and the risk of negative results is needed to inform stakeholders.

Advanced therapy medicinal products (ATMPs) are innovative products defined in the Directive 2001/83/EC (1), as amended by the ATMP Regulation 1394/2007 (2). These therapies can be either gene therapy medicinal products (GTMPs) or somatic cell therapy medicinal products (sCTMPs) or tissue-engineered products (TEPs). This class includes also combination ATMPs that are GTMP or sCTMP or TEP, combined with a medical device. A dramatic increase in the number of research on the use of these therapies in different therapeutic areas has been seen in the past few years. A new study identified 939 ATMPs clinical trials between 1999 and June 2015 (3); this reflects the increased investment of commercial and non-commercial sponsors in this field.

Randomized clinical trials (RCT) are considered the gold standard (4) for generating data to prove the efficacy, safety, and tolerability of a new therapy (5, 6). Developing new therapies and conducting high-quality RCT is expensive and resource-demanding (7). The discontinuation of RCT wastes scarce resources. Therefore, it is critical, on one hand, to minimize the risk of trials discontinuation and, on the other hand, to estimate the risk of trials discontinuation to integrate this information in the process of decisions making.

In addition, the reasons for the discontinuation of a clinical trial may vary from strategic sponsor decision (8) to poor recruitment (9) or serious adverse events (10). The premature discontinuation of trials has substantial adverse consequences not only for the patients but also the investigators; it deceives the patients and may jeopardize the patient–doctor relationships (8). The rate of discontinuation is an important part of trustful relationships that can be used to inform and reassure patients, and may increase their motivation, as well as that of investigators, to participate in trials.

The rate of discontinuation is one of the components of the risk of failure rate. This risk is an important driver of the expected net present value that informs manufacturer prioritization to invest in a portfolio of products (11).

A study by Kasenda et al. showed that the rate of discontinuation of RCT is 25% (12). No or very few ATMPs were included because the concept of ATMPs is new, and the trials included in this study were registered between 2000 and 2003; moreover, potential ATMPs were not reviewed separately. To the authors’ knowledge, there are no data on the rate of the discontinuation of ATMPs clinical trials.

The objective of our study is the estimation of the number and characteristics of discontinued ATMPs clinical trials in order to evaluate the discontinuation rate.

## Materials and methods

Data were collected by two independent researchers who retrieved withdrawn, terminated, or prematurely ended ATMPs clinical trials, referred to as ‘discontinued trials’ in this article, conducted during the time period from 1999 to June 2015 and ongoing and completed trials conducted from 1999 to June 2013 with a recent last update date (last update date after May 2014). Three clinical trials databases were searched: Clinicaltrials.gov, the International Clinical Trials Registry Platform (ICTRP) of the World Health Organization (WHO), and the European Clinical Trials Database (EudraCT). The same combinations of keywords was used for the three databases searches. (For more details on materials and methods, see the relevant article (3)).

Data extraction forms were designed using Microsoft Excel 2010 to extract the registration number, date of registration, title, status, phase, study design, target enrolment number, sponsor, disease, and last update date.

Duplications were removed, as well as therapies not classified as ATMPs. We classified the remaining trials by ATMPs type, based on the definition of ATMP provided by the European regulation EC N° 1394/2007 (2).

The data were sorted out by:

- Type of ATMPs: sCTMPs, GTMPs, TEPs, and combined ATMPs.
- Sponsor status: commercial, non-commercial. For non-commercial sponsors, the corresponding clinical trials were classified into five settings: hospital, university, institute, medical center, and government.
- Development phase: phase 1, 1/2, 2, 2/3, and 3.
- Pathology: Cancer, cardiovascular, musculoskeletal, immune system/inflammation, neurology, gastro-intestinal/diabetes, ophthalmology, pulmonology, dermatology (wounds, ulcers), and others.

We calculated the rate of discontinuation of ATMPs clinical trials using:

$$\text{Probability of discontinuation of ATMP trial}=\frac{\text{Number of discontinued trials}}{\left(\text{Number of discontinued trials}+\text{Number of ongoing and completed trials}\right)}$$

We calculated the rate of discontinuation of trials for every category of ATMPs, in every phase, and by sponsor status.

## Results

### Rate of discontinuation

Between 1999 and June 2015, 143 withdrawn, terminated, or prematurely ended ATMPs clinical trials were identified. The number of ongoing and completed clinical trials registered between 1999 and June 2013 with a recent last update date (last update date after May 2014) is 474 trials: 391 ongoing and 83 completed. Therefore, the estimated rate of discontinuation of ATMPs trials is 23.18%.

Of the discontinued trials, 71.3% are sCTMPs, 17.48% are TEPs, 9.79% are GTMPs, and 1.4% are combined products.

The rate of discontinuation of cell therapies trials is 27.35%, it is 16.28% for gene therapies, 16.34% for TEP, and 40% for combined products (Table 1).

**Table 1:** Rate of discontinuation in every category of ATMPs

	Discontinued trials	Ongoing and completed trials	Total	Rate of discontinuation (%)
Somatic cell therapies	102 (71.33%)	271 (57.17%)	373 (60.45%)	27.35
Gene therapies	14 (9.79%)	72 (15.19%)	86 (13.94%)	16.28
Tissue-engineered products	25 (17.48%)	128 (27.00%)	153 (24.80%)	16.34
Combined products	2 (1.4%)	3 (0.64%)	5 (0.81%)	40
Total	143 (23.18%)	474 (76.82%)	617 (100%)	23.18

This table shows the number of discontinued, ongoing, and completed trials and the rate of discontinuation by ATMPs category, that is, somatic cell therapies, gene therapies, tissue-engineered products, and combined products.

### Status of the trials and targeted therapeutic areas

The majority of the discontinued trials were in the early phases of development: phase 1 and 1/2 (63.63%); 30.06% were in phase 2 and 2/3; and 6.30% were in phase 3.

The rate of discontinuation is 23% for phase 1, 1/2 and phase 3; and 26.54% for phase 2 and 2/3 (Table 2).

**Table 2:** Phase of development and therapeutic areas targeted by the discontinued trials

Therapeutic area	Phase 1 and 1/2	Phase 2 and 2/3	Phase 3	Total	Discontinuation rate per disease area (%)
Cancer	56 (64.40%)	27 (31.00%)	4 (4.60%)	87 (60.84%)	43.06
Cardiology	16 (55.17%)	11 (37.93%)	2 (6.90%)	29 (20.28%)	19.20
Immunodeficiency and inflammation	4 (57.14%)	1 (14.29%)	2 (28.57%)	7 (4.89%)	10.45
Musculoskeletal diseases	2 (50.00%)	1 (25.00%)	1 (25.00%)	4 (2.80%)	6.78
Neurology	4 (80.00%)	1 (20.00%)		5 (3.49%)	11.11
Gastrointestinal diseases and diabetes	1 (33.33%)	2 (66.67%)		3 (2.10%)	8.82
Ophthalmology	2 (100%)	0		2 (1.40%)	9.52
Pulmonology	1 (100%)	0		1 (0.70%)	12.5
Dermatology	2 (100%)	0		2 (1.40%)	15.38
Others (XCGD, enzyme deficiency)	3 (100%)	0		3 (2.09%)	17.64
Total	91 (63.63%)	43 (30.06%)	9 (6.30%)	143 (100%)	–
Discontinuation rate per phase	23.04%	26.54%	23.68%	–	–

This table shows the number of discontinued trials by phase and therapeutic area.

The discontinuation rate in oncology is the highest (43%); 87 discontinued trials (60.84%) are for ATMPs targeting several cancers: 64.40% of them in phase 1 and 1/2, 31.00% in phase 2 and 2/3, and 4.60% in phase 3. The second highest discontinuation rate is for cardiology trials (19.2%), 29 discontinued cardiology trials were identified, 55.17% of them are phase 1, 1/2; 37.93% in phase 2, 2/3; and 6.90% in phase 3. The rates of discontinuation were lower for the rest of the disease areas: dermatology (15.38%), pulmonology (12.50%), neurology (11.11%), immunology (10.45%), ophthalmology (9.52%), GI diseases (8.82%), musculoskeletal disease (6.78%), and others (XCGD, enzyme deficiency) 17.64% (Table 2).

### Sponsors

Around one-quarter (26.57%) of the withdrawn, terminated, or prematurely ended trials were sponsored by commercial companies, and 73.43% were sponsored by non-commercial sponsors: 45.71% of them are universities, 30.48% institutes, 10.48% hospitals, 10.48% medical centers, and 2.86% government.

The probability of discontinuation is almost the same for commercial and non-commercial sponsors, 24.52% and 22.73%, respectively (Table 3).

**Table 3:** Rate of discontinuation by sponsor status

	Discontinued trials	Ongoing and completed trials	Total	Rate of discontinuation (%)
Commercial sponsors	38 (26.57%)	117 (24.68%)	155 (25.12%)	24.52
Non-commercial sponsors	105 (73.43%)	357 (75.32%)	462 (74.88%)	22.73
Universities	48 (45.71%)	136 (38.10%)	184 (39.83%)	26.09
Hospitals	11 (10.48%)	114 (31.93%)	125 (27.06%)	8.80
Institutes	32 (30.48%)	57 (15.97%)	89 (19.26%)	35.95
Medical centers	11 (10.48%)	32 (8.96%)	43 (9.31%)	25.58
Government	3 (2.86%)	18 (5.04%)	21 (4.54%)	14.28
Total	143 (23.18%)	474 (76.82%)	617 (100%)	23.18

This table shows the number of discontinued, ongoing and completed trials and the rate of discontinuation by sponsor status.

## Discussion

Bringing new drugs to the market is a very expensive process (13); drugs development costs are increasing to nearly $2 billion for each marketed drug (14). Firms need to do portfolio decisions based primarily on expected net present value. Amongst many factors, the trials discontinuation rate is a factor used to determine the expected net present value of a therapy, which helps the manufacturers decide about investing in a product portfolio and setting their priorities.

In addition, this information may reduce the feelings of mistrust that can complicate the difficult decision about whether the patient will join a trial (15). The patient may be willing to integrate this information when agreeing to enroll in a clinical trial, and we believe such information may be useful and should be part of informed consent. At the same time, physicians may also want to assess the risks of the discontinuation or failure of clinical trials when deciding to act as an investigator for a trial, in order to be reassured that they are committing themselves to a trial that can lead to important scientific results and is less risky than others. This information may also be part of the mix that informs decisions (8).

To our knowledge, no studies evaluated this risk for ATMPs. Our study aimed to identify the discontinued ATMPs clinical trials and evaluate the risk of discontinuation.

Between 1999 and June 2015, 143 ATMPs clinical trials were withdrawn, terminated, or prematurely ended; between 1999 and June 2013, 391 ongoing and 83 completed clinical trials started. The probability of the discontinuation of ATMPs is 23.18%. It is almost the same rate of discontinuation that was evaluated by Kasenda et al. (12) for drugs in development. Therefore, the development of ATMPs as a class may not be riskier than other therapies. However, the differences between therapeutic classes are important, and the aggregated discontinuation rate should be considered carefully.

The rate of discontinuation of ATMPs trials targeting cancer is the highest (43%), and is considerably higher than the discontinuation rate of cancer trials evaluated by Stensland et al. (16). They showed that, between 2005 and 2012, the rate of the discontinuation of cancer trials in phase 2 and 3 was 11.5%. In cardiology, the rate of discontinuation of ATMPs trials is around 20%, whereas it is 10.9% (17) for cardiology clinical trials between 2000 and 2013. It is unclear why the oncology products result in such a higher risk. It may be that the prices of oncology products are higher (18–20) than those in other therapeutic areas, and the incentives to invest in the oncology field are consequently higher. Therefore, investors and manufacturers may be open to more risk-taking for ATMPs targeting oncology, indicating that reward may be more important.

Surprisingly, the development phase does not affect the discontinuation rate. The reason is unclear. Is it related to the lowest number of studies in development in later phases or the largest number of studies completed in earlier phases, or a specificity of ATMPs, in which the discontinuation rate may be independent of development phases? This should be further explored as more products reach a later stage.

The discontinuation rates for commercial and non-commercial sponsors are the same, suggesting that the reason of discontinuation may not be financially driven. One may have anticipated that commercial organizations are more cautious and attract fewer risk takers, thus resulting in a lower risk of discontinuation rates compared to the rates of non-commercial organizations. This possibility is not supported by our findings.

This study carries some limitations. First, the reason for discontinuation is not available in the searched databases, and therefore some discontinuations may not be related to scientific/technical considerations. Second, another way to do this analysis would have been to identify all studies registered at a given time interval and follow them up to discontinuation or completion. However, this may have significantly reduced the number of eligible trials because we need to have a sufficiently long follow-up to reach either completion or discontinuation, and too many trials are still ongoing. Actually, 85% of the trials identified until June 2015 were still ongoing and 15% were completed (3). Alternatively, we limited our study to all ongoing and completed trials registered up to June 2013 and all discontinuations up to June 2015. This means that, for the most recently registered trials, we gave at least 2 years follow-up to identify a potential discontinuation, which allowed the consideration of more trials. In addition, the probability of transitioning between phases was not identified; this will be the objective of our future work. Third, although it may not occur frequently, some trials may have been discontinued trials that were not necessarily reported by the sponsor. Fourth, ATMPs trials started before the ATMPs regulation may not be easily identified, however, a recent study (3) showed that the number of ATMPs trials grew exponentially, suggesting older trials carry a low impact on the overall results. These limitations also suggest our estimated discontinuation rate may be slightly underestimated though unlikely to significantly impact the results.

## Conclusion

No failure risk rate per development phase is available for ATMPs. Discontinuation rates may prove helpful when assessing the expected net present value to support portfolio prioritization, or to persuade patients and potential investigators to participate in trials. These results carry some limitations as the rationale for discontinuation is unknown. The overall rate of discontinuation for aggregated development phase, and indications, is about 23.18%. However, it may be slightly but not significantly underestimated. Oncology products experienced the highest discontinuation rates, but discontinuation rates are not affected by the development phase of trials, unlike those for small molecules and biologics. Further research is needed regarding the reasons for discontinuation and the actual risk of failure in order to inform patients, potential investigators, manufacturers, and investors.
